# Non-Invasive Prenatal Diagnosis of Multiple Endocrine Neoplasia Type 2A Using COLD-PCR Combined with HRM Genotyping Analysis from Maternal Serum

**DOI:** 10.1371/journal.pone.0051024

**Published:** 2012-12-07

**Authors:** Hada C. Macher, Maria A. Martinez-Broca, Amalia Rubio-Calvo, Cristina Leon-Garcia, Manuel Conde-Sanchez, Alzenira Costa, Elena Navarro, Juan M. Guerrero

**Affiliations:** 1 Department of Clinical Biochemistry, The Virgen del Rocío University Hospital (IBiS/CSIC/SAS/University of Seville), Seville, Spain; 2 Department of Endocrinology, The Virgen del Rocío University Hospital (IBiS/CSIC/SAS/University of Seville), Seville, Spain; VU University Medical Center, The Netherlands

## Abstract

The multiple endocrine neoplasia type 2A (MEN2A) is a monogenic disorder characterized by an autosomal dominant pattern of inheritance which is characterized by high risk of medullary thyroid carcinoma in all mutation carriers. Although this disorder is classified as a rare disease, the patients affected have a low life quality and a very expensive and continuous treatment. At present, MEN2A is diagnosed by gene sequencing after birth, thus trying to start an early treatment and by reduction of morbidity and mortality. We first evaluated the presence of MEN2A mutation (C634Y) in serum of 25 patients, previously diagnosed by sequencing in peripheral blood leucocytes, using HRM genotyping analysis. In a second step, we used a COLD-PCR approach followed by HRM genotyping analysis for non-invasive prenatal diagnosis of a pregnant woman carrying a fetus with a C634Y mutation. HRM analysis revealed differences in melting curve shapes that correlated with patients diagnosed for MEN2A by gene sequencing analysis with 100% accuracy. Moreover, the pregnant woman carrying the fetus with the C634Y mutation revealed a melting curve shape in agreement with the positive controls in the COLD-PCR study. The mutation was confirmed by sequencing of the COLD-PCR amplification product. In conclusion, we have established a HRM analysis in serum samples as a new primary diagnosis method suitable for the detection of C634Y mutations in MEN2A patients. Simultaneously, we have applied the increase of sensitivity of COLD-PCR assay approach combined with HRM analysis for the non-invasive prenatal diagnosis of C634Y fetal mutations using pregnant women serum.

## Introduction

Multiple endocrine neoplasia type 2 (MEN2) is composed of three clinical subtypes, multiple endocrine neoplasia type 2A (MEN2A), familial medullary thyroid carcinoma, and multiple endocrine neoplasia type 2B, all of which are associated with germline mutations in the *RET* proto-oncogene. Particularly, MEN2A is an autosomal dominant hereditary syndrome caused by mutation of the tyrosine kinase c-*RET* proto-oncogene on chromosome 10q11.2 [1]. The hallmark of the disease is medullary thyroid cancer (MTC) which occurs in nearly 100% of cases [2] while pheochromocytoma (PHEO) is identified in 30–50% of patients [3], and hyperparathyroidism occurs in 20% of patients and is usually mild and manifests as only a slight elevation of serum calcium [4]. Its prevalence is approximately 1∶25.000, accounting for more than 80% of all MEN2 cases [5]. Although this disorder is classified as a rare disease the patients affected have a low life quality and a very expensive and continuous treatment.

MTC represents the first clinical manifestation of MEN2 syndrome and is the principal cause of morbidity and mortality. Early identification of MTC patients is important because of its tendency to metastasize at an early age. Although several biochemical tests were used for the early detection of MTC in the past [6], it was the discovery of the correlation between mutations of *c-RET* and MEN2 syndrome in 1993 that opened a new era in the early recognition of the patients. The DNA-based testing of the *c-RET* gene on a blood sample offers the opportunity for early identification of the *c-RET* germline mutations, thus contributing to the reduction of morbidity and mortality of MEN2 syndrome [7]. In fact, the early recognition of the mutant gene carriers makes the prevention and cure of MTC possible, by performing a prophylactic thyroidectomy before the clinical expression of the tumor including genetic counseling [8].

The discovery of circulating cell-free DNA (cfDNA) in blood plasma has led to a breakthrough in molecular diagnosis over the last ten years [9]. Different hypotheses were performed to explain the origin of cfDNA. They are supposed to derive from dead cells due to necrosis or apoptosis [10], but some authors reported the possibility of an active release from cells [11]. The presence of DNA and RNA circulating freely in the blood stream of healthy subjects can be related to activated lymphocytes and lysis of other nucleated cells or their active secretion. In patients affected by neoplastic diseases it is supposed that normal and cancer cells can: (i) detach from the tumor mass and undergo necrosis or apoptosis; or alternatively (ii) actively release nucleic acids into the blood flow. As regard to the biological significance of cfDNA, it has been suggested that metastases might develop as a result of transfection to susceptible cells in distant target organs with dominant oncogenes that circulate in plasma and are derived from the primary tumor [12], [13]. The quantity of circulating DNA is generally very low in healthy subjects (less than 5 ng/ml of plasma), while it increases (5–10 times) when considering subjects affected by neoplasic diseases [13] and in some physiologic conditions, such as pregnancy [14] as well as in benign pathologies.

During the last decade, cfDNA has received special attention because of its potential application as a non-invasive, rapid, and sensitive tool for molecular prenatal diagnosis and monitoring of fetal pathologies since cell-free fetal DNA (cffDNA) can be detected in maternal blood [15]. cffDNA contributes some 10% of the DNA in maternal plasma and could be readily detected by basic molecular techniques, being detected in maternal plasma just weeks after conception [16], [17] and is promptly cleared and disappears within 2 h of delivery [18]. As a result, cffDNA in maternal plasma appears to be a promising source of fetal genetic material for the development of non-invasive prenatal diagnosis (NIPD).

## Materials and Methods

### Participants and processing of serum samples

Twenty five patients diagnosed of MEN2A by standard methods (sequencing DNA isolated from peripheral blood leucocytes) with a C634Y mutation in *c-RET* gen (GeneBank AJ243297), located on 10q11.2, were used to study the correlation between the diagnosis by the standard method and by serum cfDNA studies. Additionally, a healthy pregnant woman with 20 weeks of pregnancy and whose husband suffered the C634Y mutation was also studied to determine whether measuring cffDNA in the serum could be used for the diagnosis of the fetal C634Y mutation. Five additional healthy subjects, negative for the C634Y mutation, were used as controls.

Blood samples of all participants were collected by venous puncture, centrifuged at 4°C, at 3.500×g for 10 min; then, immediately transferred to CryoTube Vials (Nunc, Roskilde, Denmark), and stored at -80°C until further DNA extraction. The study was approved by the Ethical Committee of the Virgen del Rocío University Hospital and has the written informed consent of all participants.

### PCR amplification and high resolution melting gene scanning

DNA was automatically extracted from 400 µL serum using the MagNa Pure Compact instrument (Roche Diagnostics, Bassel, Switzerland) with the Magna Pure Compact Nucleic Acid Isolation Kit I according to the Total NA Plasma 100 400 V3 1 extraction protocol. DNA was eluted in a final volume of 50 µL elution buffer and stored at −80°C until used for further testing. DNA from control positive patients was serially diluted into wild type DNA to the following percentages (2.5, 5, 10, 20, 25, and 50%) as described by Milbury et al [19].

For the diagnosis of MEN2A in serum using cfDNA, the real time PCR and HRM analysis were performed in the same run in a Light Cycler 480 (Roche Applied Science, Bassel, Switzerland), generating a product of 106 pb, containing the 634 position of the *c-RET* gene exon 11. Primers used for amplification and designed by the free Primer3 software were MEN Forward (5′- GGCGGTGCCAAGCCTCAC- 3′) and MEN Reverse (5′- CACCGAGACGATGAAGGAGA- 3′). PCR reactions were performed in a total volume of 20 µL in Light Cycler plaques with a reaction mixture containing 2 µL of eluted DNA, 0.4 µL each primer (10 µM), 1.6 µL MgCl_2_ (25 mM), 5.6 µL distilled water, and 10 µL Master Plus 480 HRM probes (Roche Applied Science, Bassel, Switzerland). Cycling conditions were as follows: 1 cycle of 95°C for 10 min, 48 cycles of 95°C for 5 s, 65°C for 10 s, and 72°C for 10 s. Melting analysis was at 95°C for 1 min with a rampe rate of 4.40°C/s, 40°C for 1 min with a rampe rate of 2.20°C/s, 60°C for 1 min with a rampe rate of 4.40°C/s, and increasing the temperature to 95°C with a rampe rate of 0.02°C/s reading the fluorescence during this process. The last step was one cycle of 40°C for 30 s. Result interpretation was performed using the Light Cycler 480 Software Gene Scanning program.

For non-invasive prenatal diagnosis of MEN2A using cffDNA in the maternal serum, a fast COLD-PCR (co-amplification at lower denaturation temperature PCR) followed by a HRM in the same run was performed. COLD-PCR exploits melting temperature (T_m_) differences between variant sequences and wild-type sequences. This approach uses a critical denaturation temperature (T_m_) to selectively amplify minority mutated alleles [20]. COLD-PCR reactions were performed in a total volume of 20 µL in Light Cycler plaques with a reaction mixture containing 5 µL of eluted DNA, 0.4 µL each primer (10 µM), 1.6 µL MgCl2 (25 mM), 2.6 µL distilled water, and 10 µL Master Plus 480 HRM probes (Roche Applied Science, Bassel, Switzerland). Cycling conditions were as follows: 1 cycle of 95°C for 10 min, 45 cycles of 95°C for 5 s, 65°C for 10 s, and 72°C for 10 s, and 20 additional cycles changing the denaturation temperature from 95°C to 90°C (T_m_). Then, melting analysis was performed as previously shown. Result interpretation was performed using the Light Cycler 480 Software Gene Scanning program.

The confirmation of both HRM gene scanning and COLD-PCR amplification results was performed by sequencing of the PCR products (IMEGEN, Valencia, Spain).

## Results

### Genotyping of C634Y mutation of the *ret* protooncogene by HRM analysis in serum samples

**Figure 1:**
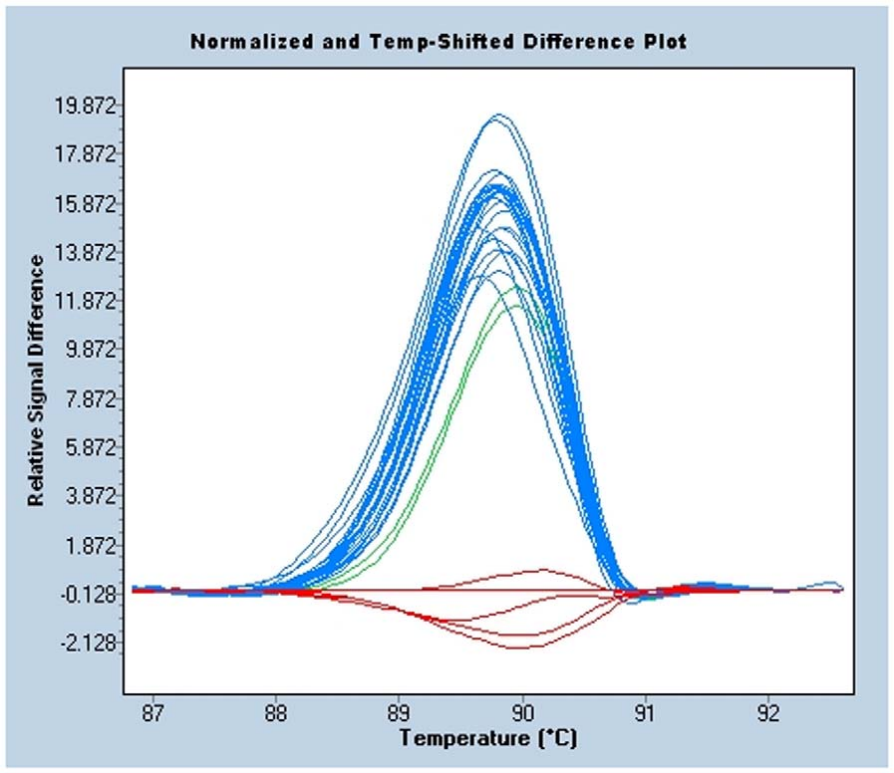
Genotyping of MEN2A in serum circulating DNA. HRM analysis of 25 MEN2A patients and 5 healthy controls (red) represented as the normalized and temperature-shifted curves converted to fluorescence different plot for analysis by the LC 480 software. HRM analysis reveals differences in melting curve shape that correlates with patients diagnosed as MEN2A vs all controls.

We have established HRM analysis in serum samples as a new primary diagnosis method suitable for the detection of C634Y mutations in exon 11 of the *c-RET* protooncogene. This variation represents a heterozygous single-base substitution (exon 11, codon 634 TGC>TAC). We have analyzed serum samples of 25 patients previously diagnosed by sequencing in peripheral blood leukocytes of MEN2A with C634Y mutation. All samples were analyzed in duplicates and plotted against 5 wild-type reference controls. After appropriate optimization, all MEN2A patients were successfully detected. The normalized melting curves, derivative plots, or fluorescence different plots could be used to distinguish the C634Y mutation from the wild-type samples (Fig. 1), showing that cfDNA isolated from serum, rather than blood cells, could be a useful tool for a heterozygous punctual mutation diagnosis.

### Non-invasive prenatal diagnosis of C634Y mutation by COLD-PCR and HRM

**Figure 2:**
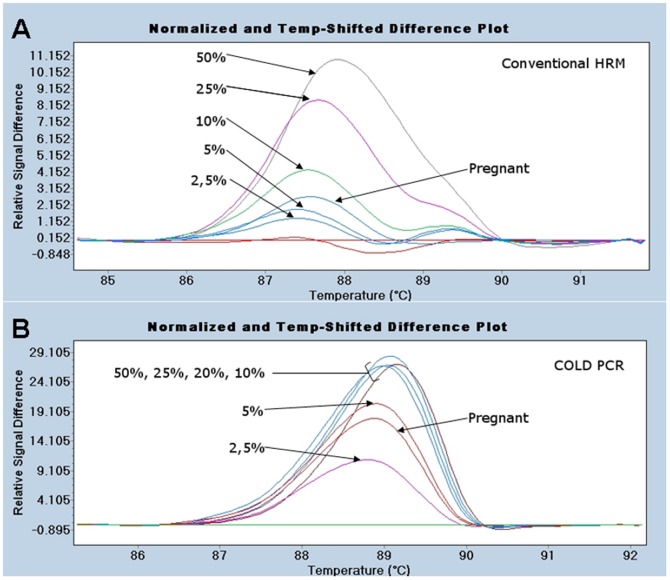
Comparison of COLD PCR and conventional HRM for non-invasive prenatal diagnosis of MEN2A. Conventional HRM (A) and Fast COLD PCR (B) analysis of one pregnant woman with a fetus carrying the MEN2A mutation, analyzed together with negative controls and serial dilutions of positive controls. Mutation-containing samples differ appreciably from wild-type melting curves. LC 480 software differences the pregnant woman carrying the MEN2A fetus from the healthy controls after conventional HRM (A, red) and COLD PCR (B, green) analysis.

We also studied a healthy pregnant woman whose couple suffered the C634Y mutation in heterozygosis and carried a fetus with the MEN2A mutation. Serum samples from both negative and positive controls were also studied. The positive control was subjected to serial dilutions as described elsewhere. All samples were studied by conventional HRM and by fast COLD-PCR followed by HRM analysis. As shown In Fig. 2, HRM (A) and COLD-PCR followed by HRM (B) analysis classified correctly the healthy and the MEN2A positive controls. All positive control dilutions differ from negative controls using both techniques. However, results from COLD PCR showed more evident differences than conventional HRM results from the lowest dilution. Moreover, COLD PCR showed the same efficiency when 50% to 10% dilutions were used as PCR template. The pregnant woman carrying the MEN2A fetus was classified as mutant, being clearly differentiated from negative controls and placed close to 5% dilution.

**Figure 3:**
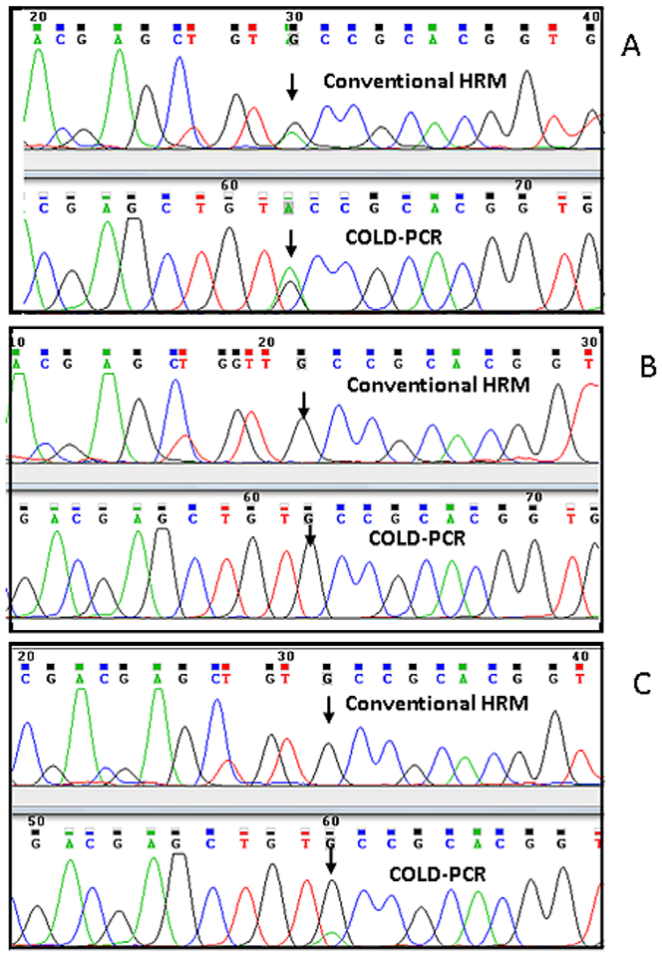
Comparison of conventional HRM and COLD PCR sequences of PCR products (G>A mutation). Sequence analysis of both HRM and COLD PCR products from a positive MEN2A with C634Y heterozygous mutation control (A), a negative healthy control (B), and the pregnant woman carrying a fetus with the C634Y mutation (C).

Both the conventional HRM and the fast COLD-PCR products were sequenced and results are shown in FIG. 3. Positive control (Fig. 3A) showed the bases guanine and adenine in the 634 position in exon 11 of *c-RET* proto-oncogene. Although both sequenced amplicons showed the mutated base in the position 634, which was observed it in a mayor proportion when COLD-PCR was performed. Negative control (Fig. 3B) shows only guanine in the same position. The COLD-PCR product sequenced from the pregnant woman with affected fetus (Fig. 3C) exhibited guanine predominantly in the position 634, showing also the presence of adenine in a quantity large enough to be detected by this method. However, when conventional HRM was performed, only the guanine was detected in this position.

## Discussion

The determination of cfDNA is becoming a useful tool for the diagnosis and management in various acute and chronic clinical disorders. Changes in levels of cfDNA have been reported in cancer, autoimmune diseases, stroke, myocardial infarction, trauma patients, renal transplantation, virus infections, and sepsis [21]–[28]. Additionally, cfDNA originated in the fetus (cffDNA) can also be detected in the plasma of the mother from the 4th week of pregnancy [29], [30] offering a potentially superb method for early NIPD of the genetic status of the fetus such as Rh and sex [31]–[33], chromosomal aneuploidies [34], and an increasing number of single gene disorders [35].

In this work, we study the cfDNA of 25 patients diagnosed with MEN2A using sequencing analysis in blood cells. At the same time, C634Y mutations were detected and, therefore, final diagnosis was reached using HRM analysis in serum. Thus, using this method, all MEN2A patients were successfully detected matching 100% the results obtained by sequencing analysis. Although the gold standard for *c-RET* mutation detection is sequencing, other methods, such as single-strand conformation polymorphism analysis, heteroduplex detection by conformation-sensitive gel electrophoresis, restriction enzyme digestion of PCR products, pyrosequencing, fluorescently labeled hybridization probes, and microarrays, have also been developed to detect *c-RET* mutations [36]. These methods, however, require additional processing of the PCR products to detect mutations, misidentify nonpathogenic polymorphisms as false positive mutations, or may target mutation hotspots missing some rare mutations increasing the rate of false negatives.

HRM analysis, however, is a rapid, closed-tube mutation scanning assay that detects sequence variation within the PCR amplicon by use of a saturating double-stranded DNA dye, and does not require post-PCR manipulation of samples or use of expensive labeled probes. This technique detects mutations between the primers, in contrast to more localized techniques, such as hybridization probes or restriction enzyme-based assays [37]. In our study, the C634Y mutation of *c-RET* proto-oncogene by HRM analysis was 100% accurate. Although not all pathogenic *c-RET* mutations were available for analysis, systematic studies of HRM detection of heterozygous point mutations within a PCR amplicon showed a sensitivity and specificity of 100% for amplicons <400 bp in size [36]. Thus, HRM analysis for mutation scanning is a rapid, cost-effective assay that requires no processing or separation steps.

In the second step, a healthy pregnant woman whose couple suffered the C634Y mutation in heterozygosis and carried a fetus with MEN2A mutation was studied. Pregnant serum sample was studied by fast COLD-PCR followed by HRM analysis, being this method able to classify properly the pregnant woman with the fetus affected from the negative controls. Thus, COLD-PCR analysis is able to identify the cffDNA found in the pregnant woman serum in estimated amounts of 1.5–10% [16]. COLD-PCR selectively enriches the low-abundance mutations within a target amplicon exploiting the small difference in amplicon melting temperature (T_m_). Just below the T_m_ there is a critical denaturation temperature (T_c_) wherein PCR efficiency drops abruptly as a result of the limited number of denatured amplicons. This difference in PCR efficiency, at specifically defined denaturation temperatures, can be used to selectively enrich minority (or low-abundance mutant) alleles throughout the course of PCR [20]. In our work, the best efficiency was obtained using the T_m_ as T_c_, observing that the pregnant woman carrying the MEN2A fetus was classified as mutant, being clearly differentiated from negative controls, and placed close to 5% dilution. Unlike the COLD PCR study, conventional HRM analysis poorly differentiated the pregnant woman from controls.

Other conventional method, such as Sanger sequencing, is typically reliable for screening germline or prevalent (clonal) somatic mutations but the sensitivity of mutation detection is typically limited to detecting approximately 10–20% mutant fractions [38]. In this way, after sequencing conventional HRM PCR product from the pregnant woman with affected fetus, we were not able to observe the presence of adenine in the position 634 in a quantity large enough to be detected by this method. However, after COLD PCR analysis, product sequencing could appreciate both bases (adenine and guanine) at this position. Other method such as pyrosequencing has a sensitivity limited to approximately 5–10% mutant in wild-type DNA [39]. Despite the increased sensitivity that this approach provides, the equipment and reagents are expensive, and it is not widely available. Additionally to both sequence-based approaches, COLD-PCR demonstrates the ability to identify mutants with much lower quantity of DNA, down to 0.1% [40].

In conclusion, we have established a HRM analysis in serum samples as a new primary diagnosis method suitable for the detection of C634Y mutations in MEN2A patients. Simultaneously, we have applied the increase of sensitivity of COLD-PCR assay approach combined with HRM genotyping analysis for the NIPD of a C634Y fetal mutation using pregnant woman serum. The results open the possibility for the use of these technologies in the prenatal diagnosis of fetal mutations inherited from the father in a safe manner for the fetus.
